# Mechanical Properties of 3D-Printed Liquid Crystalline Polymers with Low and High Melting Temperatures

**DOI:** 10.3390/ma17010152

**Published:** 2023-12-27

**Authors:** Kai S. Johann, Andreas Wolf, Christian Bonten

**Affiliations:** Institut für Kunststofftechnik, University of Stuttgart, 70569 Stuttgart, Germany

**Keywords:** 3D printing, fused filament fabrication (FFF), plastics, polymers, liquid crystalline polymers (LCP), mechanical properties, tensile testing

## Abstract

Additive manufacturing allows for the production of complex components using various types of materials such as plastics, metals and ceramics without the need for molding tools. In the field of high-performance polymers, semi-crystalline polymers such as polyetheretherketone (PEEK) or amorphous polymers such as polyetherimide (PEI) are already successfully applied. Contrary to semi-crystalline and amorphous polymers, thermotropic liquid crystalline polymers (LCPs) do not change into an isotropic liquid during melting. Instead, they possess anisotropic properties in their liquid phase. Within the scope of this work, this special group of polymers was investigated with regard to its suitability for processing by means of fused filament fabrication. Using an LCP with a low melting temperature of around 280 °C is compared to processing an LCP that exhibits a high melting temperature around 330 °C. In doing so, it was revealed that the achievable mechanical properties strongly depend on the process parameters such as the direction of deposition, printing temperature, printing speed and layer height. At a layer height of 0.10 mm, a Young’s modulus of 27.3 GPa was achieved. Moreover, by employing an annealing step after the printing process, the tensile strength could be increased up to 406 MPa at a layer height of 0.15 mm. Regarding the general suitability for FFF as well as the achieved uniaxial mechanical properties, the LCP with a low melting temperature was advantageous compared to the LCP with a high melting temperature.

## 1. Introduction

### 1.1. Fused Filament Fabrication with Conventional High-Performance Polymers

The three-dimensional printing allows for the manufacturing of complex components using different materials such as ceramics, metals and polymers without the need for expensive molding tools. Thereby, the components are produced layer by layer based on a three-dimensional computer model. In the field of polymer processing, the fused filament fabrication (FFF) is one of the most common 3D-printing techniques [1].

Research in the polymer-based FFF has so far been primarily concerned with the use of conventional polymers that exhibit a flexible macromolecular chain. The molten state of these amorphous and semi-crystalline polymers exhibits isotropic properties at equilibrium, whereby the thermodynamically favored conformation is that of a random coil [2,3]. Nevertheless, the extrusion of a polymer melt through a 3D-printing nozzle results in a certain flow-induced macromolecule alignment in the extrusion direction [4] or, in some cases, even in shear-induced crystallization [5]. However, after exiting the nozzle, the macromolecules relax. This results in the partial restoring of the orientation until the temperature falls below a certain threshold during cooling. This relaxation is macroscopically well visible in the extrudate swelling after the nozzle exit [4]. Except for the crystalline areas of semi-crystalline polymers in the solid state, conventional polymers thus tend to have less oriented structures due to the relaxation processes after exiting the printer nozzle.

In terms of the expected mechanical properties, high-performance thermoplastics such as polyetheretherketones (PEEKs) and polyetherimide (PEI) are most likely to compete with the liquid crystalline polymers (LCPs) that are considered in this present work [1,6]. Therefore, publications regarding the FFF of high-performance thermoplastics will be depicted in the following.

When processing PEEK with FFF, the extrusion temperature is around 360–400 °C with build platform temperatures of 110–120 °C [6]. In [7], a tensile strength of 56.6 MPa was achieved for 3D-printed specimens, which is more than 43% lower than the tensile strength of the injection-molded reference specimens. Scanning electron microscope (SEM) images revealed that pores located between the strands are primarily responsible for the poorer tensile properties of the printed specimens [7]. The fact that the achievable mechanical properties of 3D-printed PEEK specimens are inferior to the properties of injection-molded PEEK specimens is also shown in [7] using three-point bending tests. There, the 3D-printed specimens exhibit a 65.5% reduction in flexural strength compared to injection-molded specimens, which can be attributed to the poor interconnection of the deposited strands [7]. In [8], the tensile strength of 3D-printed PEEK specimens is also 33% lower than the tensile strength of injection-molded PEEK specimens that achieve a tensile strength of 113 MPa. The utilized FFF parameters have an extrusion temperature of 400–430 °C, a chamber temperature of 80 °C, a building platform temperature of 130 °C and an extrusion rate of 2.2 mg/s. Despite that these were optimized process parameters, the 3D-printed PEEK specimens still exhibit 14% porosity [8]. In [9], 3D-printed PEEK specimens were found to have a tensile strength between 50.6 and 74.5 MPa with a Young’s modulus of 2.6–2.8 GPa. There, the delamination between the printed PEEK layers and the pore formation is mainly attributed to thermally induced stress. This phenomenon is particularly associated with semi-crystalline thermoplastics which possess a high melting temperature [10]. In principle, the crystallinity of polymers is influenced by factors such as the cooling rate and the molecular structure, whereby PEEK exhibits a particularly complex melting and crystallization behavior due to its semi-rigid molecule structure. For instance, a so-called double-melting phenomenon can occur in PEEK with a melting peak between 200 and 330 °C and a melting peak at 330 and 340 °C [11].

In contrast to PEEK, the high-performance thermoplastic polyetherimide (PEI) is an amorphous polymer. The utilized nozzle temperatures for PEI are around 330–360 °C with build platform temperatures of 110–160 °C [6]. In [12], specimens printed with pure PEI (ULTEM^®^ 1010) exhibit a tensile strength of about 101 MPa and a Young’s modulus of 3.1 GPa. Specimens examined in [12] consist of PEI/polycarbonate (PC) blends and exhibit a reduction in tensile strength and a Young’s modulus with increasing PC content. In [13], the commercially available ULTEM^®^ 9085 is used, which is a PEI/PC blend that is associated with better flow properties than pure PEI. There, different path planning strategies with varying orientations between the deposited strand direction and the tensile test direction result in a tensile strength between 38.5 MPa and 71 MPa, a Young’s modulus of 1.8–2.5 GPa and an elongation at break between 2% and 7% [13]. The PEI/PC blend ULTEM^®^ 9085 is also used in [14] where it is revealed that the chemical smoothing of the printed specimens with gaseous chloroform only leads to a slight increase in tensile strength with both untreated and treated specimens being in the range of 50 MPa–70 MPa. In [15], ULTEM^®^ 9085 is used for 3D-printing with different path planning strategies (0°, 90°, ±45°). Again, the achieved tensile strength is between 20 and 65 MPa, the Young’s modulus is around 0.8 and 2.2 GPa, and the achieved elongation at break lays between 2% to 6% [15].

### 1.2. Fused Filament Fabrication with Liquid Crystalline Polymers (LCPs)

Due to their special molecular structure, liquid crystalline polymers (LCPs), such as the aromatic polyesters with a rigid main chain of benzene rings and ester groups, exhibit a thermodynamically stable anisotropic nematic mesophase instead of an isotropic melt. In the nematic mesophase, the macromolecules possess a preferred long-range orientation along the so-called director *n*, even in the resting state [16]. The isotropic phase is usually not reached in common LCP because LCP thermally decomposes before the ‘nematic-to-isotropic’ phase transition occurs [17]. Hence, their thermoplastic processing takes place in the anisotropic nematic mesophase and not in the isotropic melt, contrary to conventional polymers. The LCP forms a randomly oriented domain structure with a domain size in the range of approximately 10 μm [18]. The LCP orientation within one domain is along the local director *n* but may differ from domain to domain. However, by applying a shear and/or extensional flow, the domains of the nematic mesophase can be aligned macroscopically and globally, respectively, in a uniform direction [19,20,21]. Additionally, the LCP exhibit a complex defect topology and so-called disclinations that influence the molecular orientation and the texture of the LCP [18].

Lyotropic LCP such as certain aromatic polyamides only possess liquid crystalline properties in combination with a solvent [22]. In contrast, the thermotropic LCP such as specific aromatic polyesters exhibit a transition from the solid state to the nematic mesophase solely due to an increase in temperature [22].

Another group of macromolecular liquid crystals are liquid crystal elastomers (LCEs). Recently, LCEs have been thoroughly studied in the field of 3D- or 4D-printing [23,24,25,26]. Nevertheless, LCE will not be further discussed at this point since they belong to the field of functional materials and not to the field of high-strength structural materials that are primarily focused in the present work. Moreover, the printing process used for LCE does not take place via an ordinary FFF but also involves ultraviolet (UV) light-induced chemical crosslinking during the printing process.

Using thermotropic LCP in FFF is quite an unexplored research field, particularly regarding the usage of pure LCP. In contrast, several studies exist regarding the FFF of blend filaments consisting of a thermotropic LCP in combination with conventional polymers. In [27], an elaborate dual extrusion system is used to produce filament blends of polypropylene (PP) and a reinforcing thermotropic LCP. There, 3D-printing with a PP/LCP monofilament with 40 wt.% LCP at 240 °C printing temperature leads to a Young’s modulus of 2.7 GPa and a tensile strength of 37 MPa. On the contrary, a printing temperature of 290 °C, which is above the melting temperature of the utilized LCP, results in a decrease in Young’s modulus to 2.4 GPa and a decrease in the tensile strength to 21 MPa [27]. A similar dual extrusion procedure to form a blend filament is presented in [28,29], where acrylonitrile butadiene styrene (ABS) is processed as a matrix with a thermotropic LCP as reinforcement material. The especially designed dual extrusion system is used for blending since the processing temperatures of the two materials differ significantly. On the one hand, the ABS/LCP filaments with 40 wt.% LCP achieve high mechanical properties with a tensile strength of 169 MPa and a Young’s modulus of 39.9 GPa [28]. On the other hand, using the ABS/LCP filaments for the 3D-printing of specimens results in a significantly lower tensile strength of 74.9 MPa and a Young’s modulus of 16.5 GPa, even under optimized printing parameters [28]. The printing process of the ABS/LCP filament took place at temperatures of 240–270 °C to allow the melting of the ABS matrix while maintaining the solid state of the LCP phase. The lowest printing temperature results in poor adhesion between the printed layers, whereas the highest temperature results in bubbles in the printed component, which is attributed to the thermal degradation of the ABS [28]. Resembling investigations that use the filament blends of an LCP together with a polyphenylene sulfide (PPS) can be found in [29,30,31]. There, the 3D-printed PPS/LCP samples exhibit a tensile strength of 108.5 MPa and a Young’s modulus of 25.9 GPa. A combination of a carbon fiber (CF)-reinforced PEEK with a thermotropic LCP is studied in [32]. There, it is found that strong pi–pi interactions between the aromatic rings of PEEK and LCP are beneficial since they improve the crystallization behavior of PEEK and the processability of PEEK/CF composites [32].

Two wide-ranging and thorough studies regarding the FFF of pure thermotropic LCPs were published by Gantenbein et al. [33,34]. In [33] a nozzle temperature of 295 °C, a build platform temperature of 90 °C and a printing speed of 35 mm/s were used among other process parameters. It was found that the pure LCP strands exhibit a distinct core–shell structure. This phenomenon is explained by the fact that the flow within the printer nozzle causes the nematic domains to align in the flow direction. After the nozzle exit, the shell layer of the strand cools rapidly, resulting in a fixation of the highly oriented molecules [33]. On the contrary, the slower cooling of the core layer allows for a relaxation due to thermal fluctuations and thus results in a somewhat less oriented core [33]. The 3D-printed LCP specimens achieve a high tensile strength of approximately 400–500 MPa with an elongation at break of around 4% [33]. It is shown that a subsequent annealing step at 260 °C for 96 h in a nitrogen atmosphere increases the tensile strength of the specimens as well as the elongation at break. Furthermore, the toughness is almost doubled by the annealing step from 13.4 MPa to 27.6 MPa [33]. In [34], the FFF of a thermotropic LCP is performed with a novel printing technology—the so-called spin-sprinting—which combines regular, thick printing strands with thin spun fibers. These spun fibers are tenfold smaller than the regular printing strands and exhibit a significantly higher tensile strength and Young’s modulus, which is attributed to the larger fraction of a highly oriented shell layer in the thin fibers compared to a thick strand [34].

The aim of this present work is to contribute to a better understanding of how the mechanical properties of LCP samples produced via FFF are influenced by the respective printing conditions. In doing so, a commonly utilized LCP, which has a low ‘crystalline-to-nematic’ transition temperature of 280 °C, is compared to a high melting LCP that exhibits a ‘crystalline-to-nematic’ transition temperature of 330 °C. To the best of our knowledge, there does not exist any literature yet that uses an LCP with a melting temperature above 300 °C in 3D-printing. Thus, the following process parameters are examined with regard to the effect on the tensile properties: the nozzle temperature, the printing speed, the layer height and the path planning. Furthermore, the influence of a subsequent annealing step at elevated temperatures is investigated.

## 2. Materials and Methods

### 2.1. Chemical Composition of the Utilized High- and Low-Melting-Point LCPs

Two different aromatic co-polyester LCPs were used in this work. The first is Vectra^®^ A950 from the Celanese Corporation, Irving, TX, USA, which is referred to as *LCP-L* (L: low melting temperature) in the following. The *LCP-L* is a random co-polyester based on para-hydroxybenzoic acid (*p*-HBA) and 2, 6-hydroxynaphthoic acid (HNA), possessing a monomer ratio of 73/27 for HBA and HNA, respectively [35]. The second is Sumikasuper RB100 from Sumitomo Chemical Co., Ltd., Tokyo, Japan. This material will be referred to as *LCP-H* (H: high melting temperature) hereinafter. According to the online available information from Sumitomo Chemical, the *LCP-H* is primarily based on three different monomers: *p*-HBA, 4,4′-Biphenol and terephthalic acid. Compared to the *LCP-L*, this *LCP-H* does not contain naphthalene-based units (so-called crankshaft units) which reduce the melting temperature because they result in an offset on the chain axis [35,36]. Thus, the utilized *LCP-H* exhibits a higher melting temperature compared to *LCP-L*. Differential scanning calorimetry (DSC) measurements of both materials can be found in Section 3.4.3.

### 2.2. Thermal, Chemical and Mechanical Characterization Methods

Measurements with differential scanning calorimetry (DSC) as well as thermogravimetric analysis (TGA) were performed to characterize the thermal characteristics of both LCPs. A Mettler Toledo DSC 2 (Mettler-Toledo, LLC, Columbus, OH, USA) was utilized to determine the glass transition temperature (*T*_g_) and the ‘crystalline-to-nematic’ transition temperature (*T*_CN_). In the case of *LCP-L*, not all endotherms that were observed in the DSC measurements represent a ‘crystalline-to-nematic’ transition. To account for this, the term melting temperature (*T*_m_) is used in the following for endothermic events. Two heating runs and one cooling run were performed between −20 °C and 400 °C with a heating/cooling rate of 10 K/min using nitrogen (N_2_) as the purge gas. To ensure the thermal equalization, the temperature was kept constant for five minutes at −20 °C before starting the heating run and for two minutes at 400 °C before starting the cooling run. Aluminum crucibles were used and the sample mass was always around 10–15 mg. A Mettler Toledo TGA850 (Mettler-Toledo, LLC, Columbus, OH, USA) was used to determine the degradation temperature. One heating run was performed from 30 °C until 950 °C with a heating rate of 10 K/min, 50 mL/min synthetic air and an initial sample mass of around 15 mg.

Fourier-transform infrared spectroscopy (FT-IR) was performed by using a Tensor 27 FT-IR spectrometer, Bruker Optics GmbH & Co. KG, Ettlingen, Germany with an L-alanine-doped triglycine sulfate (DLaTGS) detector. The scan ranged from 4000 cm^−1^ to 400 cm^−1^ with a resolution of 4 cm^−1^ utilizing the attenuated total reflection (ATR) technique.

The mechanical properties were characterized using the uniaxial tensile test machine Universalprüfmaschine ZT20 from Zwick Roell AG, Ulm, Germany. The used load cell has a maximum force of 20 kN. The clamping length was set to 50 mm. The tensile samples had a rectangular shape with a “length × width × height” of 120 mm × 10 mm × 2 mm for 0° samples and 90 mm × 10 mm × 2 mm for 90° samples. Six samples were characterized for each printed set. The test speed amounts to 1 mm/min for determining the Young’s modulus and increased to 5 mm/min after a strain value of 0.25% was exceeded.

### 2.3. Fused Filament Fabrication Procedure

Both LCPs were processed via FFF using an E3D Toolchanger motion system from E3D-Online, Chalgrove, United Kingdom, possessing several component adaptions. The utilized high-temperature hot-end for FFF was from Slice Engineering, Gainesville, FL, USA, and allowed printing temperatures of up to 500 °C with a 0.4 mm diameter titanium nozzle. The layer height varied between 0.10 and 0.20 mm, the printing speed varied between 25 and 45 mm/s and the nozzle temperature varied between 300 and 440 °C. The utilized LCP filaments exhibited a diameter of around 1.75 mm. The *LCP-H* exhibited a certain diameter irregularity due to mass fluctuations that appeared during the filament production with a single-crew extruder from Collin Lab & Pilot Solutions GmbH, Maitenbeth, Germany. The mass fluctuations were primarily caused by a poor trickling behavior of the powdery *LCP-H* from the hopper and could be avoided in future work using a melt pump, for instance. On the contrary, the *LCP-L* filament had a very uniform diameter. G-codes for the path planning at FFF were created with the slicer software ideaMaker 4.1.1 from Raise 3D Technologies, Inc., Irvine, CA, USA. The tensile test samples were printed with two different path planning strategies, namely 0° and 90°. Here, 0° means that the printing lines were deposited parallel to the tensile direction and 90° means that the printing lines are perpendicular to the tensile direction. The 90° direction was chosen to quantify the adhesion between different layers, whereas the 0° direction should result in the highest mechanical properties since the orientation of LCP molecules is expected to be parallel to the tensile direction in this case. The build platform temperature was set to 120 °C and a bonding agent was used to improve the adhesion of the printed samples on the build platform.

## 3. Results and Discussion

It should be noted that the assumption of a better molecular orientation, which is made in some cases in the following, will be analyzed in more detail by means of wide-angle X-ray scattering (WAXS) measurements that allow for the calculation of the order parameter *S*, which is a quantitative measure for molecular orientation. The respective WAXS results will be published elsewhere due to its large extent.

**Figure 1:**
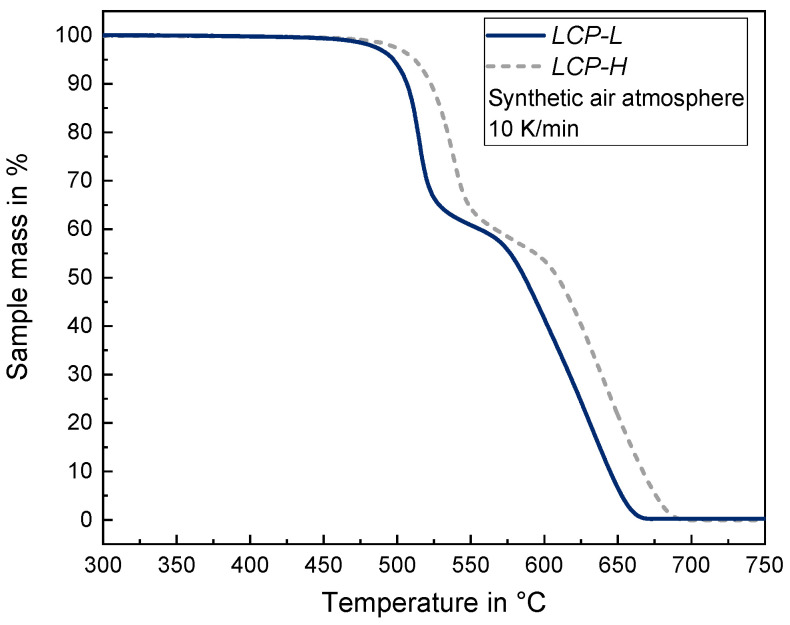
TGA curves of *LCP-L* and *LCP-H* in order to examine the thermal degradation behavior.

The TGA results are depicted in Figure 1. Qualitatively speaking, both LCPs exhibit a similar two-step thermal decomposition behavior. The decomposition of *LCP-L* starts at around 466 °C whereas the decomposition of *LCP-H* starts at a slightly higher temperature of approximately 480 °C. A significant sample mass reduction of 5% is observed at 497 °C for the *LCP-L* and at 512 °C for the *LCP-H*. The TGA results are in good accordance with the findings in [37], where a two-step decomposition of the *LCP-L* was observed in an air atmosphere. On the contrary, a nitrogen atmosphere resulted in a one-step decomposition. Using a coupled TGA-FTIR analysis, ref. [37] revealed that the primary degradation products are carbon dioxide (CO_2_) and carbon monoxide (CO) in both the air and nitrogen atmospheres. The oxygen (O_2_) present in the air atmosphere participates in the decomposition reaction and lowers the required activation energy *E_a_* for thermal decomposition [37].

### 3.1. Influence of the Printing Temperature

The sample name for printed samples will obey the following pattern: “Printing direction”—“nozzle temperature in °C”—“printing speed in mm/s”—“layer height in mm”. Thus, “L–300–45–0.15” means that the samples were printed with 0°, at a nozzle temperature of 300 °C, with a printing speed of 45 mm/s and with a layer height of 0.15 mm. *L* represents linearly printed samples (respectively, 0° samples) and *P* represents perpendicularly printed samples (respectively, 90° samples).

In the case of *LCP-L*, the printing temperature was set to 300 °C, 320 °C, 340 °C, 400 °C and 420 °C with the lowest chosen printing temperature being around 20 °C above the melting temperature. In the case of *LCP-H* the printing temperature was set to 350 °C, 400 °C, 420 °C and 440 °C with the lowest chosen printing temperature also being around 20 °C above the melting temperature. In both cases, using a lower temperature that is even closer to the melting temperature was omitted in order to obtain a reasonable layer adhesion.

**Figure 2:**
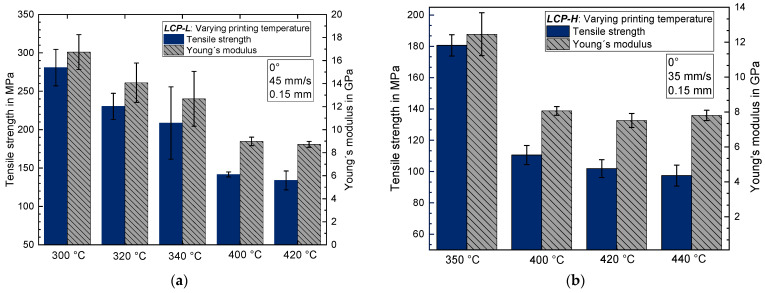
Tensile strength and Young’s modulus as a function of printing temperature (**a**) for *LCP-L* and (**b**) for *LCP-H*.

Figure 2 shows that the tensile strength decreases for both LCPs with the increasing printing temperature in the case of 0° samples. The *LCP-L* exhibits a tensile strength of 281 MPa ± 24 MPa and the *LCP-H* exhibits a tensile strength of 181 ± 7 MPa for the lowest printing temperature. The Young’s modulus also decreases with increasing printing temperature. For the *LCP-L* the Young’s modulus decreases from 16.7 ± 1.5 GPa to 12.7 ± 2.4 GPa by raising the printing temperature from 300 °C to 340 °C. The *LCP-H* exhibits the same trend but with lower values. There, the Young’s modulus decreases from 12.4 ± 1.2 GPa to 7.8 ± 0.3 GPa by increasing the printing temperature from 350 °C to 440 °C. Nevertheless, the values for the three printing temperatures of 400–440 °C are close to each other, indicating that the decreasing tendency of tensile strength and Young’s modulus runs into a plateau by exceeding a certain threshold temperature. On the other hand, increasing the printing temperature results in an increase in the elongation at break, which is raised from 2.7 ± 0.2% (at 300 °C) to 3.5 ± 0.6% (at 340 °C) in the case of *LCP-L*.

Using the *LCP-L* allows for a higher tensile strength and Young’s modulus compared to the *LCP-H*, particularly at temperatures that are close to (20 °C above) the melting point of the respective LCP. Moreover, even at the same printing temperature, the *LCP-L* still exhibits a slightly higher tensile strength and Young’s modulus than the *LCP-H*. For a printing temperature of 400 °C, the *LCP-L* has a tensile strength of 142 ± 3 MPa and a Young’s modulus of 9.0 ± 0.4 GPa, whereas the *LCP-H* only exhibits a tensile strength of 111 ± 6 MPa and a Young’s modulus of 8.0 ± 0.2 GPa.

The DSC measurements of printed samples with varying nozzle temperatures showed no significant effect of the printing temperature on the endothermic melting peaks (peak temperature as well as melting enthalpy). Thus, a varying crystallinity is excluded for having a major effect on the mechanical properties in this case. It is thus very likely that increasing the printing temperature leads to a worse molecular orientation along the printing direction and thus to a lower tensile strength and Young’s modulus for the 0° samples.

**Figure 3:**
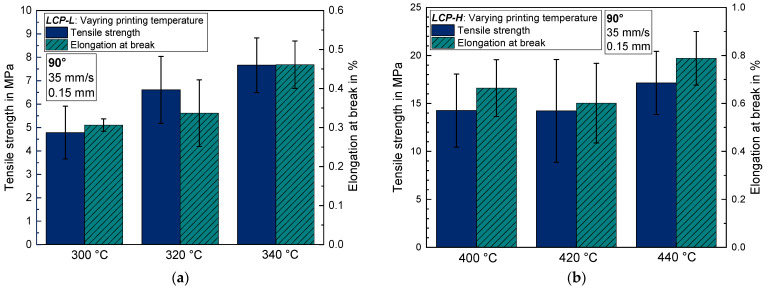
Tensile strength and elongation at break for 90° samples as a function of printing temperature (**a**) for *LCP-L* (**b**) and for *LCP-H*.

In contrast to the observation for 0° samples, an increasing printing temperature results in an increasing tensile strength for 90° samples (see Figure 3). The maximum tensile strength amounts to 17.1 ± 3.3 MPa for *LCP-H* at 440 °C and to 7.7 ± 1.2 MPa for *LCP-L* at 340 °C. Like tensile strength, the elongation at break also increases by increasing the printing temperature for 90° samples. Nevertheless, the elongation at break is very low and amounts to 0.31–0.46% and 0.60–0.79% for *LCP-L* and *LCP-H*, respectively.

Increasing the printing temperature results in a better layer adhesion and thus to an increasing tensile strength for the 90° samples. This is because higher temperatures lead to a higher thermally induced molecular motion and thus to a favorable welding behavior of the neighboring deposited LCP strands. The positive temperature effect on layer adhesion is also well known for conventional polymers such as PLA [38] and PEEK [39], where a better layer-to-layer adhesion is achieved using a heated chamber. To accomplish a good layer, adhesion is particularly challenging for the printed LCP due to its anisotropic diffusion characteristics, since translational diffusion perpendicular to the polymer chain axis is negligible compared to the diffusion parallel to the chain axis [40]. Thus, the circumstance that the molecular orientation lies parallel to the LCP–LCP interface at printing is disadvantageous for physical diffusion across the interface and thus results in quite poor welding quality between individual strands. Contrary, a molecular orientation perpendicular to the interface would result in a significantly faster diffusion across the interface and a better layer adhesion [40].

It can be concluded that there is a certain trade-off regarding the printing temperature for both *LCP-L* and *LCP-H*. Increasing the printing temperature significantly reduces the tensile strength along the printing direction, probably due to a worse molecular orientation, but increases the layer adhesion due to a generally higher molecular mobility and because the molecules partly lose their parallel orientation to the interface, which also facilitates the physical interdiffusion. Hence, the printing temperature should be chosen according to the desired application of the 3D-printed part. If there is primarily a unidirectional load and the layer adhesion in the y and z directions is of no importance: lower printing temperatures are favorable.

**Figure 4:**
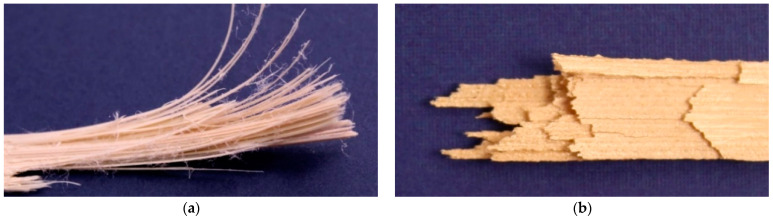
Fracture pattern of (**a**) *LCP-L* (L–300–45–0.15); and of (**b**) *LCP-H* (L–420–35–0.15).

**Figure 5:**
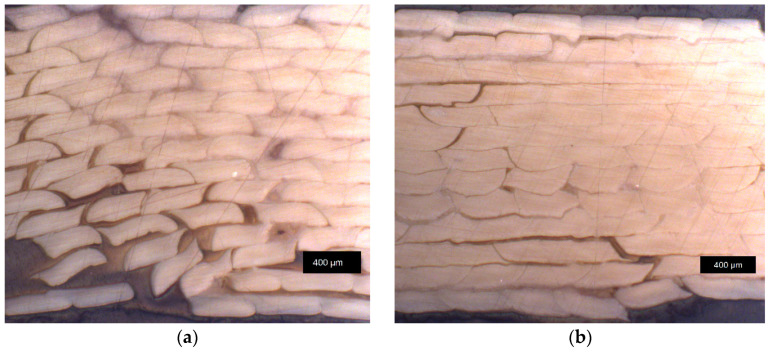
Light microscope image of *LCP-L* at a printing temperature of (**a**) 300 °C (L–300–45–0.15); and of (**b**) 340 °C (L–340–45–0.15). Samples were analyzed after tensile testing, close to the fracture plane.

Exemplary pictures of fractured tensile testing samples are depicted in Figure 4. A fibrous material structure is very pronounced in the case of *LCP-L* (Figure 4a). The *LCP-H* (Figure 4b) shows a less pronounced fibrous structure compared to the *LCP-L*. However, higher printing temperatures generally lead to a diminishing fibrous structure for both LCP. A light microscope image of *LCP-L* can be found in Figure 5 with a printing temperature of 300 °C and 340 °C. The examined plane is perpendicular to the tensile direction and both samples were analyzed after tensile testing. The fracture results in a loss of cohesion both between strands within a layer (y direction) and from layer to layer (z direction). Nevertheless, a higher printing temperature results in less strand detachment compared to a lower printing temperature due to the improved welding behavior.

### 3.2. Influence of the Printing Speed

**Figure 6:**
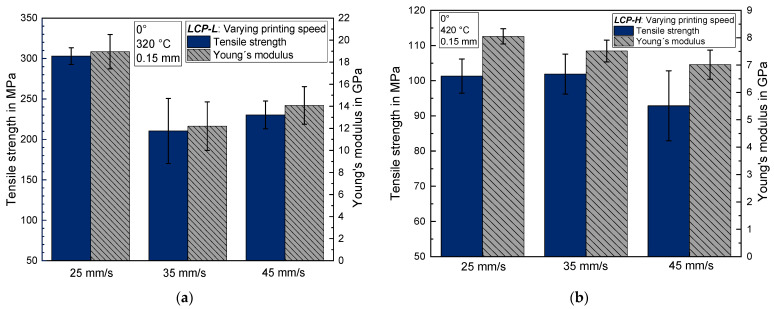
Tensile strength and Young’s modulus as a function of printing speed (**a**) for *LCP-L*; and (**b**) for *LCP-H*.

The influence of printing speed on the tensile strength and Young’s modulus is depicted in Figure 6a for *LCP-L* and in Figure 6b for *LCP-H*. In the case of *LCP-L*, the highest printing speed of 45 mm/s results in a tensile strength of 230 ± 17 MPa, and the lowest printing speed of 25 mm/s results in a tensile strength of 303 ± 10 MPa. The results of *LCP-H* show no significant influence of the printing speed on the tensile strength for the chosen temperature. However, the highest printing speed results in a slightly lower tensile strength compared to both other printing speeds. The Young’s modulus shows the same tendency as the tensile strength for both LCPs.

This result is contrary to the expected behavior because the authors assumed a higher tensile strength for an increasing printing speed due to an increasing shear rate of the melt and thus a better molecular orientation in the printing direction. Nevertheless, an observation that can be made for both LCP is that the highest printing speed leads to the highest standard deviation, whereas the samples that were printed with the lowest speed exhibit the lowest standard deviation. Thus, it can be assumed that higher printing speeds lead to a macroscopically worse printing quality. The assumption that higher printing speeds lead to a worse printing quality was also shown in [41] for a wood fiber/polylactic acid composite (WPC) filament. There, higher printing speeds lead to a lower sample density and to an uneven surface of the part with a narrower width of printed layers [41]. In [33], it was shown that already small angle deviations between tensile load and strand deposition direction lead to a substantial reduction in tensile strength and Young’s modulus for LCP. Hence, the observed behavior in this case might be a superposition of two effects, namely a better molecular orientation due to higher shear rates but also a macroscopically worse printing accuracy and quality, respectively.

Additionally, the quantitative analysis of light microscope images of *LCP-L* using the open architecture software ImageJ, version 1.54g, was conducted on 20 randomly chosen strands for a sample that was printed with 25 mm/s (L–320–25–0.15) and with 45 mm/s (L–320–45–0.15). The mean strand width amounts to 419 ± 34 µm for the 25 mm/s sample and to 413 ± 53 µm for the 45 mm/s sample. The mean strand height amounts to 153 ± 13 µm for the 25 mm/s sample and to 170 ± 17 µm for the 45 mm/s sample. Thus, the 45 mm/s sample exhibits a higher standard deviation for both the strand width and height and a higher strand height compared to the lower printing speed. This observation can also account for the lower tensile strength and Young’s modulus at the higher printing speed (see Section 3.2).

### 3.3. Influence of the Layer Height

**Figure 7:**
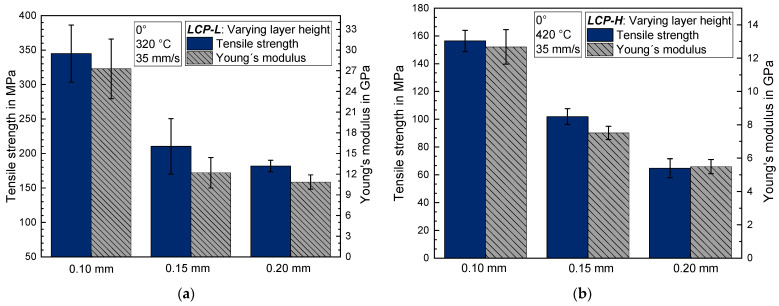
Tensile strength and Young’s modulus as a function of layer height (**a**) for *LCP-L* and (**b**) for *LCP-H*.

Reducing the layer height from 0.20 mm to 0.10 mm leads to a significant increase in the tensile strength as well as the Young’s modulus for both LCPs (see Figure 7). The tensile strength of *LCP-L* increases from 182 ± 8 MPa to 345 ± 41 MPa and the Young’s modulus increases from 10.9 ± 1.0 GPa to 27.3 ± 4.3 GPa when decreasing the layer height. The *LCP-H* exhibits a qualitatively similar behavior but with lower absolute values. Contrary to the clear trend for the tensile strength and Young’s modulus, the elongation at break is nearly constant for *LCP-H* (around 1.8–2.0%) but increases for *LCP-L* from 1.9% to 3.3% when the layer height is increased from 0.10 mm to 0.20 mm. The increasing tensile strength and Young’s modulus might be explained by the fact that LCP fibers typically possess a highly oriented skin and a less oriented core structure [42,43]. A decreasing layer height thus results in a higher skin-to-core ratio and thus in higher mechanical properties according to [33]. However, we assume that this current skin-to-core ratio explanation is not fully accurate, and a subsequent publication will deal with this issue in more detail.

**Figure 8:**
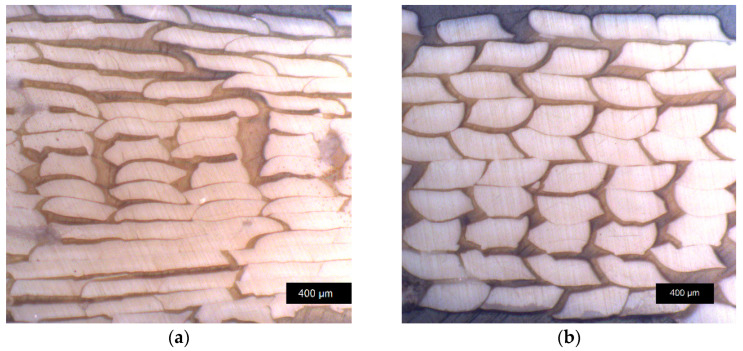
Light microscope image of *LCP-L* at a layer height of (**a**) 0.10 mm (L–320–35–0.10) and of (**b**) 0.20 mm (L–320–35–0.20). Samples were analyzed after tensile testing, close to the fracture plane.

Light microscope images of fractured *LCP-L* samples with layer heights of 0.10 mm and 0.20 mm can be found in Figure 8a and Figure 8b, respectively. It should be emphasized that the visible strand separation is only caused by the fracture and that the non-tested samples exhibit good cohesion within the whole sample. According to the quantitative analysis of 20 randomly chosen strands, the mean strand width amounts to 414 ± 44 µm for the 0.1 mm sample and to 419 ± 25 µm for the 0.2 mm sample. The mean strand height amounts to 101 ± 12 µm for the 0.1 mm sample and to 193 ± 13 µm for the 0.2 mm sample. The previously defined layer height and the real layer height of the printed sample are thus in good agreement for both cases.

The generally lower tensile strength and Young’s modulus of the *LCP-H* (see also Section 3.1) can partly be explained by taking additional wide-angle X-ray scattering (WAXS) measurements into account, which were conducted with two tensile test specimens. In the case of *LCP-H*, the specimen L–350–35–0.15 exhibited an order parameter of around 0.48, whereas in the case of *LCP-L* the specimen L–300–35–0.20 exhibited an order parameter of around 0.66. This means that the *LCP-L* exhibits a higher molecular order in the printing direction than the *LCP-H* at a printing temperature that is around 20 °C above the melting temperature of the respective LCP. This is particularly remarkable since the examined *LCP-L* specimen was printed with a higher layer height than the *LCP-H* specimen and higher layer heights usually lead to a decreasing order parameter [33].

Thus, the *LCP-L* is beneficial for high uniaxial properties since the molecular order in the printing direction is higher. This observation is somewhat against the expected behavior since the *LCP-L* possesses crankshaft units in its polymer chain that lead to an offset of the chain axis and thus to a lower melting temperature. Thus, a worse molecular order and lower mechanical properties would intuitively be expected for the *LCP-L*. Whether this unexpected observation is caused by the fact that the *LCP-H* has to be processed at higher absolute temperatures than the *LCP-L* or whether it is caused by its molecular structure cannot be conclusively be clarified at this point. Additionally, it should be noted that the molecular weight distribution of both LCPs is unknown, which could also partly be responsible for the observed differences.

### 3.4. Influence of a Subsequent Annealing Step at Elevated Temperatures

#### 3.4.1. Mechanical Characterization of Annealed Samples

For the *LCP-L*, an annealing temperature of 260 °C was chosen, since it is well known that annealing close to the melting point leads to a significant increase in the mechanical properties of LCPs [44,45]. For the *LCP-H*, the annealing temperature should be 310 °C in order to provide the same 20 °C distance to the melting point, as it is the case for *LCP-L*. However, 300 °C was the maximum temperature available for the utilized vacuum furnace and thus 300 °C was chosen as the annealing temperature for the *LCP-H*. Hence, the annealing of the *LCP-L* takes place closer to (20 °C below) the melting temperature, whereas the annealing for the *LCP-H* is slightly more distant to (30 °C below) its melting temperature. The vacuum was applied during annealing in order avoid undesirable oxidation reactions.

**Figure 9:**
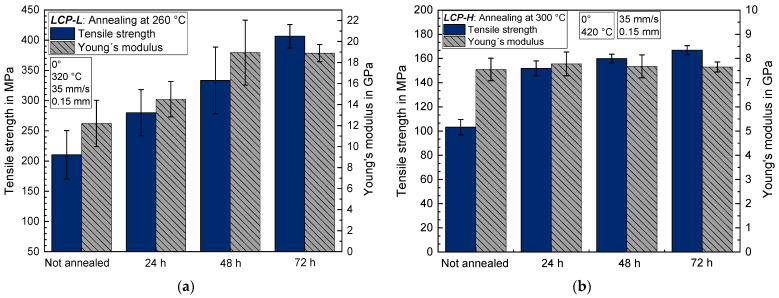
Tensile strength and Young’s modulus as a function of annealing time (**a**) for *LCP-L* and (**b**) for *LCP-H*.

**Figure 10:**
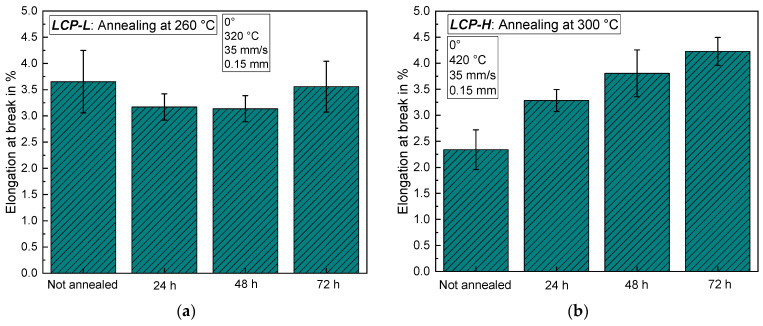
Elongation at break as a function of annealing time (**a**) for *LCP-L* (**b**) and for *LCP-H*.

The influence on tensile strength and Young’s modulus of a subsequent annealing step at elevated temperatures under vacuum is depicted in Figure 9a for *LCP-L* and in Figure 9b for *LCP-H*. For *LCP-L*, longer annealing times lead to a steady increase in tensile strength, which rises from 210 ± 40 MPa without annealing to 406 ± 19 MPa after 72 h of annealing at 260 °C. The Young’s modulus also increases from 12.2 ± 2.2 GPa to 18.9 ± 0.8 GPa after 72 h. In the case of *LCP-H*, the tensile strength rapidly increases within the first 24 h from 103 ± 6 MPa to 152 ± 6 MPa. After that, the tensile strength only increases slowly up to 167 ± 4 MPa after 72 h at 300 °C. The Young’s modulus is unaffected by the annealing and remains at around 7.6 ± 0.5 GPa independent of the annealing time, which is contrary to the observation for *LCP-L*. Similarly, the observation differs for both LCPs regarding the elongation at break. In the case of *LCP-L*, the elongation at break remains roughly constant between 3.1 and 3.6%, whereas the *LCP-H* exhibits a steady increase from 2.3% to 4.2% (see Figure 10).

A conclusive explanation for these unequal behaviors between both LCPs during annealing cannot be given at this point. However, the following chapters deal with elucidating the principal mechanisms that may be partly responsible for the general trend of increasing mechanical properties during annealing.

**Figure 11:**
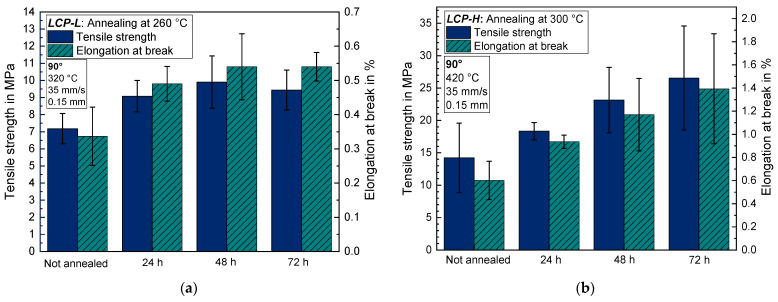
Tensile strength and elongation at break for 90° samples as a function of annealing time (**a**) for *LCP-L* and (**b**) for *LCP-H*.

The 90° samples also exhibit an increasing tensile strength and elongation at break during annealing for both LCPs, indicating an improving layer adhesion even at annealing temperatures that are below the melting temperature of the respective LCP (Figure 11). The tensile strength increases after 24 h from 7.2 ± 0.9 MPa to 9.1 ± 0.9 MPa and remains nearly constant during the residual annealing time for the *LCP-L*. On the contrary, the *LCP-H* shows a steady increase in the tensile strength from 14.2 ± 5.3 MPa to 26.5 ± 8.0 MPa after 72 h. The standard deviation for 90° samples is quite high for both LCPs. The higher layer adhesion of *LCP-H* results not necessarily from material properties itself but is probably caused by the significantly higher printing temperatures that were chosen for the *LCP-H*.

#### 3.4.2. Sample Density of Annealed Samples and Optical Microscopy

**Figure 12:**
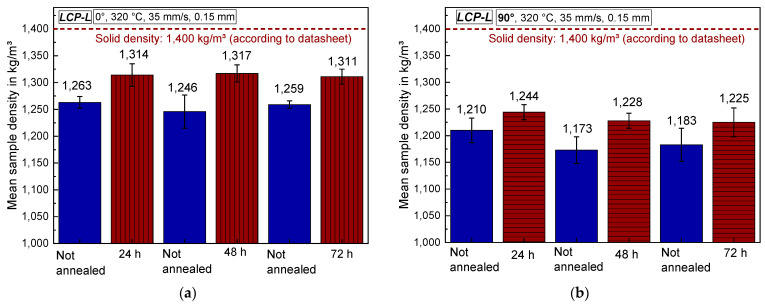
Mean density (**a**) for 0° and (**b**) for 90° samples as a function of the annealing time for *LCP-L*.

The mean sample density before and after annealing was determined by simply weighing the samples, measuring the sample dimensions with a caliper, calculating the volume, and dividing the determined mass and volume. It can be seen in Figure 12 that the density for both 0° and 90° samples increases by approximately 3–6% due to annealing. The volume decreases during annealing, whereas the mass remains constant. In the case of 0° samples, the sample length increased but the width and height decreased. In the case of 90° samples, the length and height decreased, whereas the width remained nearly constant. The solid density of *LCP-L* amounts to 1400 kg/m^3^ according to the datasheet and the unannealed 0° samples exhibit a density of approximately 1250 kg/m^3^. Hence, the total porosity of unannealed samples amounts to around 11% and to 6% after annealing.

**Figure 13:**
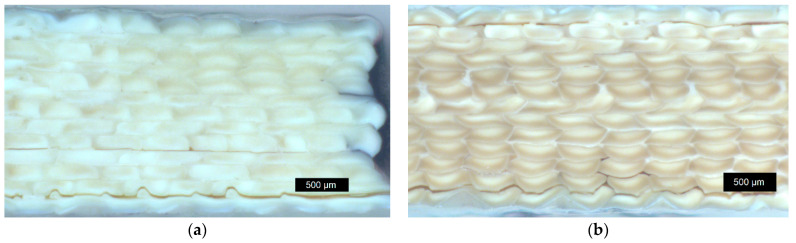
Macroscopic image of *LCP-L*: (**a**) before annealing (L–320–35–0.15); and (**b**) after annealing at 260 °C for 72 h (L–320–35–0.15–72 h). The sample was analyzed after tensile testing but with a certain distance away from the fracture plane.

**Figure 14:**
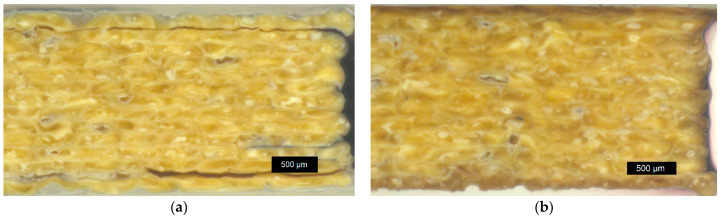
Macroscopic image of *LCP-H*: (**a**) before annealing (L–420–35–0.15) and (**b**) after annealing at 300 °C for 72 h (L–420–35–0.15–72 h). The sample was analyzed after tensile testing but with a certain distance away from the fracture plane.

Macroscopic images of *LCP-L* before annealing and after annealing for 72 h at 260 °C can be found in Figure 13a and Figure 13b, respectively. Since the *LCP-L* was printed at low temperatures, individual strands are visible. On the contrary, the strands of *LCP-H* (Figure 14) show a better coalescence due to the higher printing temperatures. The material color changed during annealing from a bright, yellow ochre to a more dark, brown ochre.

**Figure 15:**
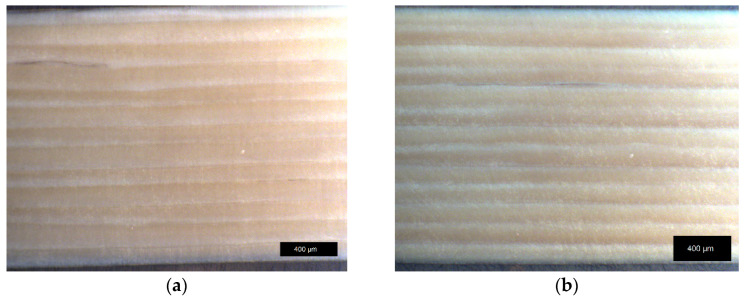
Light microscope image of 90° *LCP-L* samples: (**a**) before annealing (P–320–35–0.15); and (**b**) after annealing at 300 °C for 72 h (P–320–35–0.15–72 h). Samples were analyzed after tensile testing.

It can be concluded that the annealing had no significant influence on the macroscopic printing structure. Effects like an increasing contact area formation between individual strands (or a neck growth analogous to the so-called two-sphere sintering model from ceramics) were not observed for both LCP. Light microscope images of 90° *LCP-L* samples are shown in Figure 15. The strand deposition lies in the horizontal direction in this case. Like the 0° samples, these samples also show no macroscopically visible change with respect to the contact area between the individual layers during annealing, despite the increasing tensile strength and layer adhesion (see Figure 11).

#### 3.4.3. DSC Measurements of Annealed Samples

**Figure 16:**
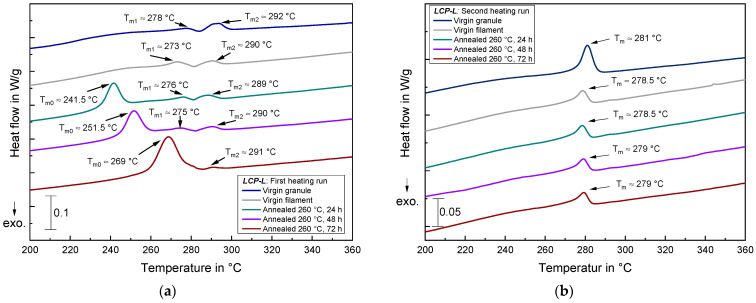
DSC measurements of *LCP-L* samples that were subject to different annealing times: (**a**) for the first heating run; and (**b**) for the second heating run. Curves are vertically shifted for the sake of clarity.

In order to explain the increasing tensile strength, Young’s modulus and sample density during annealing, DSC measurements were performed for virgin and annealed samples. Figure 16a depicts the first heating run for the *LCP-L* and Figure 16b shows the second heating run. Both virgin granule and virgin filament show two very small endothermic peaks at approximately 273–278 °C (T_m1_) and at 290–292 °C. On the contrary, the annealed samples exhibit three endothermic peaks with an additional peak (T_m0_) emerging at lower temperatures. The peak temperature of this endothermic event significantly increases during annealing from 241.5 °C at 24 h to 269 °C at 72 h. In the latter case, the peak of T_m0_ merges with the small peak of T_m1_. The totalized enthalpy for all observed endothermic events amounts to 6.8 J/g for the virgin filament, to around 9.6 J/g for the 24 h and 48 h samples and to 10.3 J/g for the 72 h sample. Thus, annealing leads to a certain increase in transition enthalpy and to the emergence of an additional endothermic event whose peak temperature shifts to higher temperatures for longer annealing times.

This observation is in good agreement with [44], where the endothermic event of T_m1_ is assigned to the conversion of an orthorhombic molecular arrangement into a pseudo-hexagonal arrangement and the endothermic event of T_m2_ is assigned to the ‘crystalline-to-nematic’ transition. In [44], the emergence of a low-temperature endothermic event was also observed (in the present paper T_m0_), whose transition temperature also increases during annealing. They concluded that the new endothermic peak is due to a conformational rearrangement of the copolymer into reorganized HBA-rich regions, which results from an entropy-driven molecular movement in order to match similar conformational sequences [44]. This phenomenon is accompanied by the displacement of HNA moieties into a less ordered phase [44]. This previous observation might also apply to the behavior in this work. However, it should be noted that the annealing temperature and time (240 °C and 20–130 min) are lower in [44] than the chosen annealing parameters in the present work (260 °C and 24–72 h). Thus, the results cannot be compared one-to-one and further studies are necessary to prove the exact mechanism in the present case.

Nevertheless, the appearance of an additional endothermic peak strongly indicates the emergence of a generally better molecular order (and superstructure, respectively) during annealing. and is thus in good accordance with the increasing tensile strength, Young’s modulus and density (see Figure 9). By taking the second heating run (Figure 16b) into account, it becomes obvious that the molecular superstructure that emerges during annealing can be eliminated for the *LCP-L*. In the second heating run, only one similar endothermic event is observed for all samples, with a melting temperature around 279–281 °C and a transition enthalpy of approximately 3.0–4.4 J/g.

**Figure 17:**
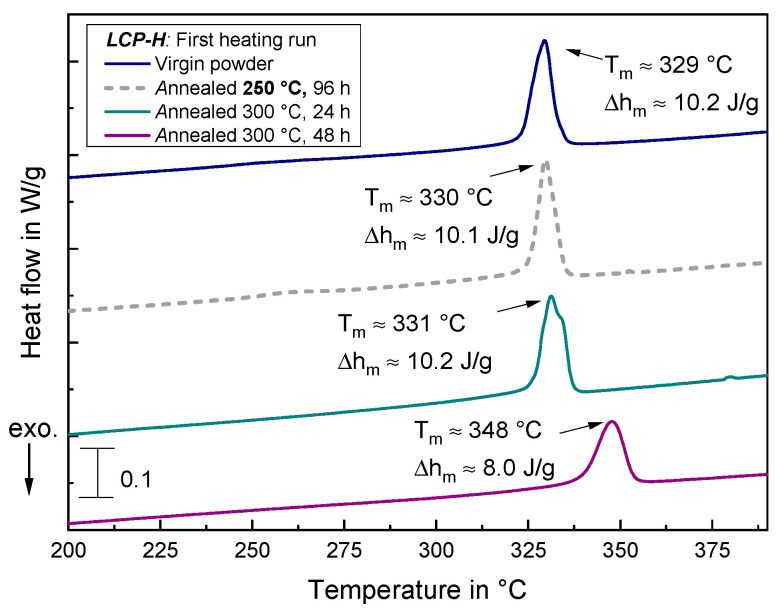
DSC measurements of *LCP-H* samples that were subject to different annealing times for the first heating run. Curves are vertically shifted for the sake of clarity.

Figure 17 illustrates the DSC results for virgin and annealed *LCP-H* samples. Contrary to the *LCP-L*, the *LCP-H* does not exhibit a newly emerging endothermic peak but only possesses one endothermic peak for all samples. At a low annealing temperature of 250 °C, the peak temperature and transition enthalpy do not change with regard to the virgin sample, even for a long annealing time of 96 h, and remain constantly around 330 °C and 10 J/g, respectively. At a high annealing temperature of 300 °C, the peak temperature T_m_, however, shifts to higher temperatures and amounts to 348 °C after 48 h of annealing. On the contrary, the enthalpy decreases to approximately 8 J/g after 48 h. A similar phenomenon, namely an increasing melting temperature in combination with a decreasing transition enthalpy was also observed in [45].

For the second heating run, the *LCP-H* exhibits the same qualitative behavior as the *LCP-L*, meaning that all heating curves are quasi-identical, independent of the annealing time. In the second heating run, the endothermic peak is also very small for all samples with a melting enthalpy of approximately 4.3 J/g and a peak temperature of 316 °C. Hence, the second heating run is not depicted for the *LCP-H* since there is no additional gain in knowledge compared to the behavior of *LCP-L*.

One mechanism that is also used in the literature to explain the increasing melting temperature due to annealing is that of so-called interchain transesterification reactions (ITR). They are known to occur for LCPs during annealing slightly below its melting temperature [46]. According to [46], the ITR can only occur in existing crystallites, leading to a sequence ordering which results in an improved packing, a higher density and a more ordered structure. The initial random-copolymer is thereby converted to a certain extent to a multiple block-copolymer [47]. On the contrary, the annealing of LCPs above the melting temperature (in the nematic melt phase) can lead to the randomizing reactions that lower the transition temperature [46].

Regarding the increase in tensile strength for 90° samples (Figure 11), two mechanisms are conceivable for the apparently increasing interpenetration of LCP molecules across different print layers, namely physical diffusion and chemical diffusion by ITR. Since physical diffusion is slow compared to chemical interdiffusion by ITR across a polymer–polymer interface [48], it is conceivable that interdiffusion by ITR might play a role at short annealing times, whereas physical diffusion might become more and more pronounced at long annealing times.

Whether both mechanisms of entropy-driven sequence ordering and ITR take place cannot be proven in our case, since other methods like nuclear magnetic resonance (NMR) spectroscopy and WAXS would be necessary. However, these are two possible explanatory approaches from the literature to explain the increasing tensile strength and Young’s modulus during annealing.

Additionally, it should be noted that the conducted examinations are not exhaustive since the annealing might also affect the material in a way that it is not detectable with the applied methods. For instance, in order to examine whether the annealing has an influence on the domain or defect structure (texture), an additional polarized light microscopy had to be performed. Especially for the *LCP-H*, it is likely that other mechanisms play a role since, in this case, the DSC measurement does not indicate a change in the endothermic peak after 24 h annealing (Figure 17), whereas this is exactly the time step which resulted in the highest increase in tensile strength and Young’s modulus for linearly printed *LCP-H* samples (Figure 9b).

It can be concluded that different mechanisms seemingly take place during annealing for the *LCP-L* and *LCP-H*. Since both LCPs significantly differ in their behavior, it becomes obvious that not only the temperature and time of annealing play an important role for the occurring mechanisms, but also the chemical structure of the material itself.

#### 3.4.4. IR Measurements of Annealed Samples

**Figure 18:**
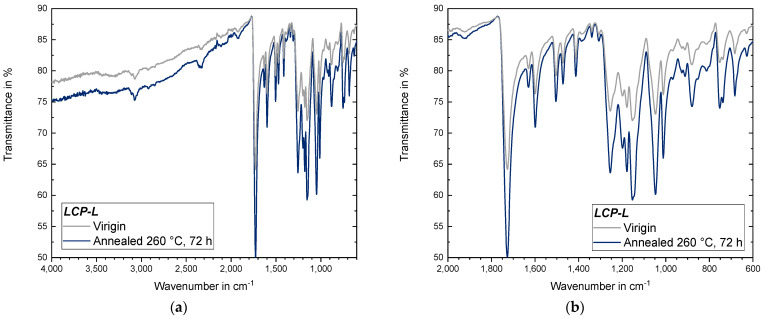
IR measurement of an *LCP-L* sample that was subject to annealing: (**a**) for the entire recorded range of 4000–400 cm^−1^; and (**b**) with a zoom to the 2000–400 cm^−1^ region.

**Figure 19:**
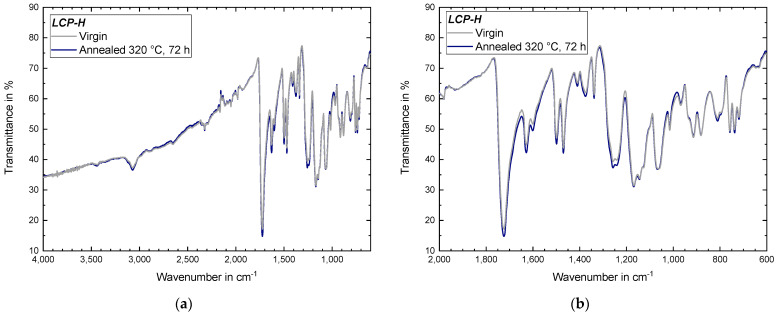
IR measurement of an *LCP-H* sample that was subject to annealing: (**a**) for the entire recorded range of 4000–400 cm^−1^; and (**b**) with a zoom on the 2000–400 cm^−1^ region.

IR measurements were performed for both *LCP-L* (Figure 18) and *LCP-H* (Figure 19) before and after annealing in order to examine whether significant chemical changes occur. According to [49], the stretching of the ester carbonyl results in a pronounced band at 1750 cm^−1^ which is found for both *LCP-L* and *LCP-H*. The stretching of C–H bonds within aromatic rings leads to a broad band at 3076 cm^−1^ [49], which is also visible for both LCPs. The broad –OH stretching band around 3550–3200 cm^−1^ is only weakly pronounced and nearly not visible. The ring vibrations of aromatic rings (here, *LCP-H* as well as *LCP-L*) lead to bands at 1600–1500 cm^−1^ and naphthalene rings (here, only *LCP-L*), resulting in two additional ring vibration bands at approximately 1634 cm^−1^ and 1473 cm^−1^ [49], which is also in good accordance with the measured data.

The mentioned ITR process (Section 3.4.3) is not visible in the IR measurements because the transesterification is only accompanied by an exchange of functional groups, where the amount of functional groups before and after the reaction remains identical [50]. The IR measurements of both LCPs also did not indicate the occurrence of chemical reactions between terminal groups during annealing at 260 °C under vacuum. Particularly, an increase in the ester (–COO–) band was expected if terminal hydroxy groups (–OH) would react with terminal carboxyl group (–COOH) to additional ester bonds, which would be accompanied by the cleavage of H_2_O. Thus, the increase in mechanical properties is primarily attributed to the increasing molecular order (emergence of an additional endothermic event and an increasing melting temperature) rather than by chemical reactions between terminal hydroxy and carboxyl groups, which is contrary to findings in [33] where annealing was performed at 270 °C. It also seems statistically rather difficult that terminal groups can react in a significant amount, since LCPs possess rigid rod-like macromolecular chains with a molecular weight (*M_w_*) of around 38,000 g/mol and a chain length of 200 nm [51], whilst annealing was performed in the solid state with low molecular mobility.

Because the sensitivity of the conducted IR measurements is limited, further investigations are, however, necessary for a final exclusion of the occurrence of chemical reaction between terminal hydroxy and carboxyl groups. For example, gel permeation chromatography (GPC) measurements could be conducted to determine the molecular weight distribution before and after annealing. Furthermore, methods like determining the acid number could be applied to investigate the amount of terminal carboxyl groups. Since LCPs exhibit a very good chemical resistance and are only soluble in a few specific solvents (and usually at elevated temperatures), both methods were not applied in the present work. This circumstance also results in the fact that, for LCP, virtually no GPC results are available in literature and, if at all, the molecular weight is derived from intrinsic viscosity data [52].

## 4. Conclusions and Outlook

### 4.1. Conclusions

This study investigated the process–structure–property relationships of a low- and a high-melting LCP during 3D-printing via FFF. The printed samples are highly anisotropic in nature, with high values in the strand deposition direction but significantly worse values perpendicular to the strand deposition direction. For both LCPs, a higher tensile strength and Young’s modulus was achieved by reducing the nozzle temperature, the layer height and the printing speed. There is a trade-off regarding nozzle temperature since temperatures close to the melting temperature result in high unidirectional tensile strength, whereas increasing the temperature leads to a better layer adhesion. At temperatures close to the melting point, the mechanical properties strongly depend on the nozzle temperature. However, by exceeding a certain threshold temperature, the tensile strength is only slightly affected by a varying temperature. Reducing the layer height from 0.20 mm to 0.10 mm proved to be very beneficial, probably due to a better molecular orientation in the tensile direction. Higher printing speeds lead to a decreasing tensile strength and Young’s modulus, assumingly due to a worse printing accuracy. Annealing at elevated temperatures increased both the tensile strength and Young’s modulus for both LCPs. The sample density as well as the transition temperature also increased during annealing, indicating that annealing leads to a further improved packing and ordering of crystalline, respectively, superstructure regions. It is likely that the exact molecular mechanism for this phenomenon differs between the utilized low- and high-melting-temperature LCPs due to the differences in their chemical structure and the different annealing temperatures. Annealed samples achieved a tensile strength of up to 406 MPa and a Young’s modulus of 18.9 GPa at 0.15 mm layer height and 35 mm/s printing speed. Utilizing a lower layer height and slower printing speed is expected to result in even higher values. Regarding the general suitability for FFF as well as the achieved uniaxial mechanical properties, the LCP with a low melting temperature was advantageous compared to the LCP with a high melting temperature.

### 4.2. Outlook

Further studies will primarily focus on the quantification of the orientation behavior of the LCP during printing, particularly by means of wide-angle X-ray scattering (WAXS) studies. Within the course of these studies, the filament will be subjected to different degrees of drawing in order to examine whether a highly drawn LCP filament will also result in a higher molecular orientation and in higher uniaxial mechanical properties of the printed samples.

Moreover, the poor layer adhesion still poses a challenge. Further studies will utilize a heated building chamber as well as a “direct annealing system” (which blows hot air onto the deposition area) in order to investigate whether a better layer adhesion can be achieved by means of these methods, without deteriorating the high uniaxial properties in an excessively pronounced way.

## Data Availability

Data are contained within the article.
